# Sacubitril/valsartan ameliorates tubulointerstitial fibrosis by restoring mitochondrial homeostasis in diabetic kidney disease

**DOI:** 10.1186/s13098-024-01284-1

**Published:** 2024-02-10

**Authors:** Xing-Jian Zhang, Cong-Cong Liu, Zuo-Lin Li, Lin Ding, Yan Zhou, Dong-Jie Zhang, Yao Zhang, Shu-Ting Hou, Rui-Xia Ma

**Affiliations:** 1 Department of Nephrology, Affiliated Hospital of Qingdao University, Qingdao, Shandong China; 2 Institute of Nephrology, Zhong Da Hospital, Southeast University School of Medicine, Nanjing, Jiangsu China; 3 Department of Nephrology, Minda Hospital Affiliated to Hubei Minzu University, Enshi, Hubei China

**Keywords:** Diabetic kidney disease, Sacubitril/valsartan, Tubulointerstitial fibrosis, Mitochondria, Sirt1, PGC1α

## Abstract

**Background:**

Tubulointerstitial fibrosis plays an important role in the progression of diabetic kidney disease (DKD). Sacubitril/valsartan (Sac/Val) exerts a robust beneficial effect in DKD. However, the potential functional effect of Sac/Val on tubulointerstitial fibrosis in DKD is still largely unclear.

**Methods:**

Streptozotocin-induced diabetic mice were given Sac/Val or Val by intragastric administration once a day for 12 weeks. The renal function, the pathological changes of tubule injury and tubulointerstitial fibrosis, as well as mitochondrial morphology of renal tubules in mice, were evaluated. Genome-wide gene expression analysis was performed to identify the potential mechanisms. Meanwhile, human tubular epithelial cells (HK-2) were cultured in high glucose condition containing LBQ657/valsartan (LBQ/Val). Further, mitochondrial functions and Sirt1/PGC1α pathway of tubular epithelial cells were assessed by Western blot, Real-time-PCR, JC-1, MitoSOX or MitoTracker. Finally, the Sirt1 specific inhibitor, EX527, was used to explore the potential effects of Sirt1 signaling in vivo and in vitro.

**Results:**

We found that Sac/Val significantly ameliorated the decline of renal function and tubulointerstitial fibrosis in DKD mice. The enrichment analysis of gene expression indicated metabolism as an important modulator in DKD mice with Sac/Val administration, in which mitochondrial homeostasis plays a pivotal role. Then, the decreased expression of Tfam and Cox IV;, as well as changes of mitochondrial function and morphology, demonstrated the disruption of mitochondrial homeostasis under DKD conditions. Interestingly, Sac/Val administration was found to restore mitochondrial homeostasis in DKD mice and in vitro model of HK-2 cells. Further, we demonstrated that Sirt1/PGC1α, a crucial pathway in mitochondrial homeostasis, was activated by Sac/Val both in vivo and in vitro. Finally, the beneficial effects of Sac/Val on mitochondrial homeostasis and tubulointerstitial fibrosis was partially abolished in the presence of Sirt1 specific inhibitor.

**Conclusions:**

Taken together, we demonstrate that Sac/Val ameliorates tubulointerstitial fibrosis by restoring Sirt1/PGC1α pathway-mediated mitochondrial homeostasis in DKD, providing a theoretical basis for delaying the progression of DKD in clinical practice.

## Introduction

Diabetic kidney disease (DKD), a common and severe microvascular complications of diabetes mellitus (DM), is the leading cause of end-stage renal disease [1]. Increasing evidence demonstrated that DKD is characterized by mesangial expansion, glomerulosclerosis, accumulation of extracellular matrix, tubular atrophy and tubular interstitial fibrosis [2]. Although angiotensin receptor blockers and sodium-glucose cotransporter 2 inhibitors significantly delay the progression of DKD [3], current therapies have limited effectiveness on the progression to end-stage renal disease. Therefore, it is urgent to develop novel therapeutic approaches to prevent or reverse the progression of DKD.

Compelling evidence indicates that tubulointerstitial fibrosis, which is main secondary to tubule injury, is predictive of the progression of DKD [4]. Recently, Zhan et al. [5] found that aberrant changes of mitochondrial morphology were observed in tubules of patients with DKD. Meanwhile, preclinical studies have shown that mitochondrial dysfunction is not only a key instigator of tubule injury but also a critical mediator in the pathophysiology of progression of DKD [6]. We recently also demonstrated that the response of tubular mitochondria to metabolic insult provokes the development of renal tubulointerstitial fibrosis [7]. Further, disruption of mitochondrial homeostasis under pathological conditions results in mitochondrial reactive oxygen species (ROS) production, energy insufficiency and leakage of the mitochondrial DNA, which further disturb mitochondrial and cellular homeostasis in a deleterious loop [8]. Thus, targeting mitochondrial homeostasis has emerged as an attractive approach to delay the progression of tubulointerstitial fibrosis.

Sacubitril/valsartan (Sac/Val) is a first-in-class angiotensin receptor-neprilysin inhibitor (ARNI) that has been recommended in clinical practice guidelines to treat patients with hypertension or heart failure [9]. Growing evidence suggested that Sac/Val provides great target organ protection. For instance, Sac/Val was proven to have cardiovascular protection effects [10]. Recently, Nishio et al. also indicated that Sac/Val ameliorated renal tubulointerstitial injury [11]. Meanwhile, it has been demonstrated that Sac/Val could attenuate proteinuria and glomerulosclerosis, improve tubulointerstitial injury in DKD mice model [12–14]. However, the exact mechanisms underpinning Sac/Val dependent renal protection effects require elucidation.

Previously, Sac/Val was found to exert organ protective effects through inhibiting self-DNA-activated cGMP-AMP synthase-stimulator of interferon genes signaling. Considering the key effect of Sac/Val on mitochondrial homeostasis, we hypothesized that Sac/Val plays a critical role in tubulointerstitial fibrosis by improving the mitochondria function in DKD. In this study, we demonstrate that Sac/Val ameliorates tubulointerstitial fibrosis by restoring Sirt1/PGC1α pathway mediated mitochondrial homeostasis. Thus, our findings provide theoretical basis for delaying the progression of DKD in clinical practice.

## Materials and methods

### Animals

Eight-week-old male C57BL/6J mice (*n* = 28; Nanjing Tande Biotechnology Co., Ltd, China) were used for the experiments. The mice were housed in an animal care facility (20 ± 1 ℃, relative humidity 45 ~ 65%) and had free access to food and water under a 12 h light/dark cycle. Four mice were housed in each cage. Twenty mice were divided into 2 groups: control group (*n* = 5) and diabetic group (*n* = 15). The sample size of each group was decided according to previous studies using STZ-induced diabetic mice [15]. To establish the type 1 DM model, the mice of diabetic group were intraperitoneally administered a low dose of streptozotocin [STZ, 50 mg/(kg·d)] dissolved in citrate buffer for 5 days. The control group were intraperitoneally administered citrate buffer. After 2 weeks, mice with fasting blood glucose (FBG) above 16.7 mmol/L were regarded as the established DM model. After excluding animals that were not successfully modeled, the diabetic group randomly divided into 3 groups: DKD group (*n* = 4), Val group (*n* = 4) and Sac/Val group (*n* = 4). Correspondingly, 4 mice in the control group were randomly selected for the next experiments.

Val [30 mg/(kg·d), NOVARTIS, Beijing, China] for the Val group, Sac/Val [60 mg/(kg·d), NOVARTIS, Beijing, China] for the Sac/Val group or vehicle (normal saline) of equal volume for the DKD group were administered gastric gavage once a day (14). After 12 weeks treatment, body weights (BW) and FBG were recorded. Then, metabolic cages were used for 24-hour urine collection. Subsequently, the mice were sacrificed to collect the blood and kidneys samples. Mass of left kidneys was weighed after sacrifice, and the kidney hypertrophy index was calculated by the ratio of kidney weight (KW) and BW.

For EX527 administration, another 8 STZ-induced diabetic mice were divided into 2 groups: Sac/Val group (*n* = 4) and Sac/Val + EX527 group (*n* = 4). The Sac/Val + EX527 group were given EX527 (20 mg/kg of EX527 diluted in 0.5% dimethyl sulfoxide) by intraperitoneal injection twice daily, and Sac/Val group were given vehicle (0.5% dimethyl sulfoxide) by intraperitoneal injection [16]. After 12 weeks treatment, the measurements and sample collection were done as mentioned above.

Complete randomization was used for the grouping of mice and the determination of treatment order. The analyses on samples were conducted by researchers blinded to treatments.

### Cell culture and treatment

The human proximal tubular cell line HK-2 was cultured in MEM medium containing 10% FBS and 1% penicillin–streptomycin at 37 ℃ in a humidified 5% CO_2_ atmosphere. After serum starvation for 24 h, HK-2 cells were divided into four groups: HG group (high glucose, 35 mmol/L), HG + Val group (HG + Val 0.01 µmol/L), HG + LBQ/Val group (HG + LBQ657/Valsartan 0.01 µmol/L; LBQ657, the active metabolite of sacubitril, Sigma-Aldrich, Shanghai, China), and HG + LBQ/Val + EX527 group (HG + LBQ657/Valsartan 0.01 µmol/L + Sirt1 inhibitor EX527). NC group (normal control, glucose 5.5 mmol/L) and HM group (NC + mannitol 25 mmol/L) were used as control groups. After treatment of 48 h, the cells were collected and molecular biological experiments were further performed.

### Renal function measurement

Serum creatinine (Scr) concentration was measured with a sarcosine oxidase creatinine assay kit (Jiancheng, China) and blood urea nitrogen (BUN) level was measured according to instructions of a urea assay kit (Jiancheng, China). Urinary creatinine concentration, N-acetyl-β-d-glucosamine-dase (NAG) and microalbumin level were quantified according to procedures of assay kits (Jiancheng, China), respectively. Then, 24-hour urinary albumin-to-creatinine ratio (ACR) were calculated.

### Histology

The renal cortical specimens were fixed in 10% paraformaldehyde and embedded in paraffin. Thin sections of tissues were created for periodic acid-Schiff base (PAS) and Masson’s trichrome staining. Histological images were visualized using an inverted microscope and analyzed using Image J software. Tubular injury was scored semiquantitatively by an observer in a blinded manner. Images of at least 20 cortical fields of PAS-stained sections were examined for each group. Tubular injury score was defined as follows: Score 0: no tubular injury; Score 1: <10% of tubules injured (tubular dilation, tubular atrophy, tubular cast formation, sloughing of tubular epithelial cells or loss of the brush border and thickening of the tubular basement membrane); Score 2: 10–25% of tubules injured; Score 3: 25–50% of tubules injured; Score 4: 50–74% of tubules injured; Score 5: >75% of tubules injured [17]. To assess tubulointerstitial fibrosis, the area of fibrosis in Masson trichrome-stained sections were measured using Image J software. Images of at least 20 randomly selected cortical fields for each group were evaluated blindly by an observer.

### Mitochondrial morphology assessment

To determine the mitochondrial morphology in tubular cells of mice kidney, tissues were fixed in 2.5% glutaraldehyde. The samples were then immersed in 1% osmium tetroxide. Then the samples were dehydrated with different acetone concentrations and made into ultrathin Sects. (50–70 nm) after embedding. They were then stained with uranyl acetate and lead citrate. Finally, they were observed by transmission electron microscopy (TEM) at 80 Kv. The longitudinal length (major) and equatorial length (minor) of mitochondria were measured using Image J to quantify the mitochondrial morphology in the renal tubular epithelial cells (RTECs) of mice kidney. Then the aspect ratio (AR) and form factor (FF) were calculated as follows: Aspect ratio = major axis/minor axis; Form factor = (Pm^2^)/(4πAm), where Pm is the perimeter and Am is the area of the mitochondria. The morphology of at least 110 mitochondria was assessed for each group.

To determine mitochondrial morphology of cells, the cultured cells were incubated with 0.1 Mm MitoTracker Red CMXRos (Thermo Fisher) at 37℃ for 20 min. Then, the cells were washed 3 times with PBS and incubated in growth medium. Fluorescence intensity was observed using a confocal microscope and analyzed using Image J.

### Western blot

The cells or kidney tissues were lysed in RIPA lysis buffer (Servicebio, China), and protein concentration was detected by bicinchoninic acid assay kits (Beyotime, China). Proteins were separated using 10% sodium dodecyl sulfate-polyacrylamide gel electrophoresis and transferred onto polyvinylidene fluoride membranes (Millipore, USA). The membranes were then sealed with NcmBlot blocking buffer (NCM Biotech) for 15 min and incubated overnight at 4 ℃ with primary antibodies against Kidney Injury Molecule-1 (KIM-1, 1:1000, MA5-28211, Invitrogen), Collagen 1 (1:1000, sc-59,722, Santa Cruz), α-SMA (1:1000, sc-53,142, Santa Cruz), Tfam (1:1000, ab272885, Abcam), COX IV; (1:5000,11242-1-AP, Proteintech), Sirt1 (1:1000, ab110304, Abcam), PGC1α (1:5000, 66369-1-Ig, Proteintech), or β-actin (1:10000, sc-47,778, Santa Cruz). After incubation with horseradish peroxidase-conjugated goat anti-mouse or anti-rabbit IgG (1:3000, Cell Signaling, USA) for 1 h at room temperature, the blots were detected with the chemiluminescence advanced system (GE Healthcare).

### RNA isolation and real-time PCR

The TriZol reagent (Vazyme, China) was used to extract total RNA from HK-2 cells according to product specifications, and HiScript III RT SuperMix (Vazyme, China) was used to reverse transcribe mRNA. Then, PCR was conducted using a 7300 Real-time PCR detection system (Applied Biosystems, USA) with ChamQ Universal SYBR Qpcr Master Mix (Vazyme, China). The data were normalized to the expression of β-actin, and the relative expression of the target genes was calculated using the 2^−ΔΔCT^ method.

### Immunohistochemical staining and immunofluorescence

For immunohistochemical staining, 2 Μm thick paraffin-embedded kidney sections were incubated with primary antibodies against Tfam (1:1000, ab272885, Abcam) overnight at 4 ℃, followed by PBS washing and incubation with biotin-conjugated goat antirabbit IgG for 30 min at room temperature. Immunoreactivity was detected using diaminobenzidine reagent (Solarbio). The sections were subsequently stained with hematoxylin and covered with neutral resin. Images were obtained by the optical microscope (Leica Microsystems). Image analysis and quantification were performed using Image J.

For immunofluorescence staining, HK-2 cells were incubated with primary antibodies against Sirt1 (1:1000, ab110304, Abcam). After washing with PBS, the cells were incubated with fluorescence conjugated secondary antibodies. The Olympus optical microscope was used to detect the images.

### Mitochondrial function assessment

Mitochondrial membrane potential (MMP) was detected using mitochondrial membrane potential assay kit with JC-1 following the manufacturer’s protocol (Beyotime, China). To measure mitochondrial ROS, HK-2 cells were incubated at 37℃ with fresh media containing 5 Mm MitoSOX Red mitochondrial superoxide indicators (Thermo Fisher) for 10 min. Then, the cells were observed by confocal microscopy.

### Statistical analysis

Data are expressed as means ± SEM. The significance of the differences in mean values between multiple groups was examined by analysis of variance (one-way ANOVA). To compare between every two groups, the LSD t-test was used. All statistical analyses were performed using GraphPad Prism 8.0. *P* < 0.05 was considered statistically significant.

## Results

### Sacubitril/valsartan improved renal function in DKD mice

**Fig. 1:**
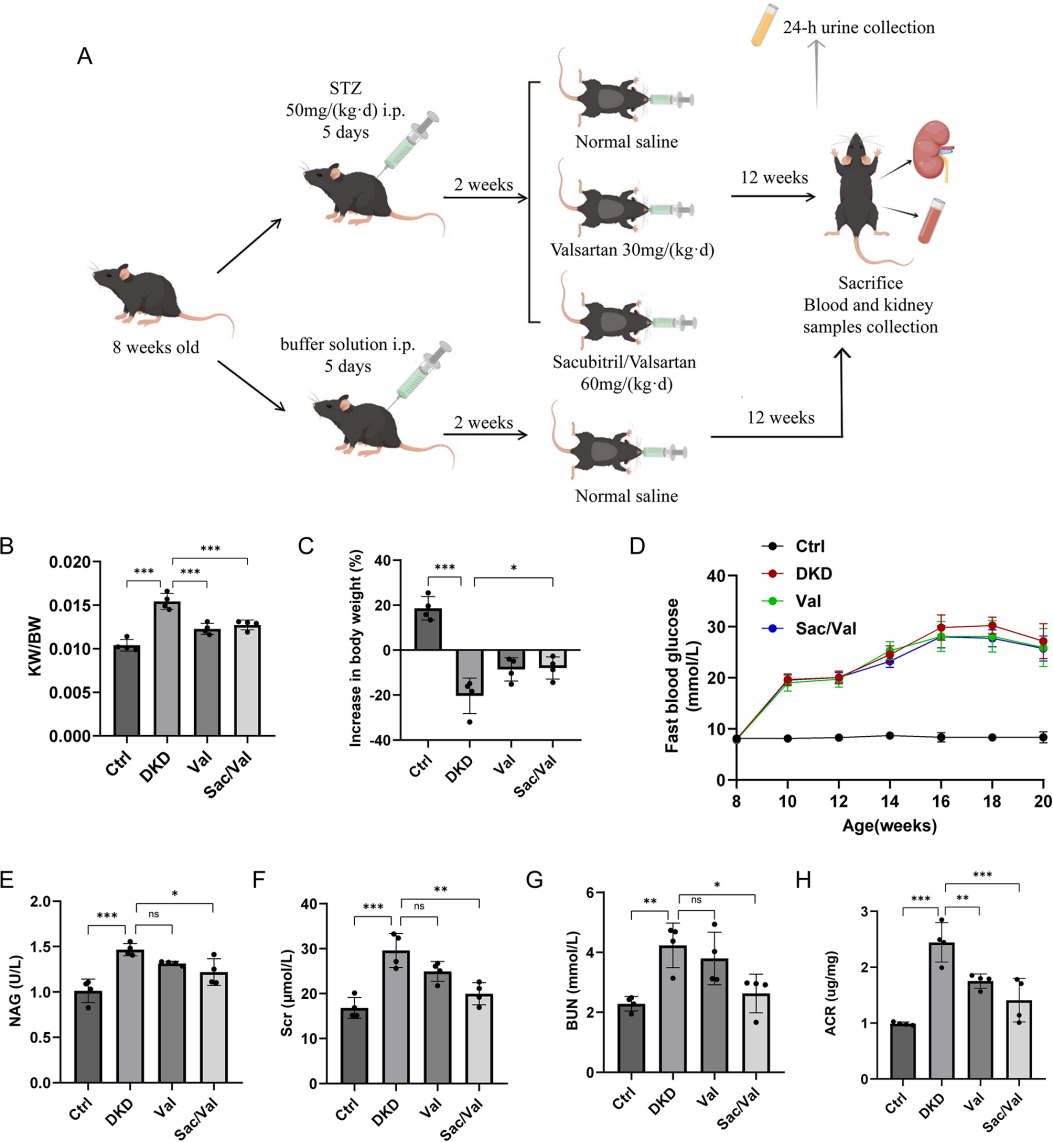
Primary metabolic parameters and biochemistry of blood and 24-hour urine in diabetic mice. **(A)** Diagram illustrating the experimental design (by Figdraw). **(B)** Kidney weight/body weight. **(C)** The percentage of increase in body weight at the end of treatment. **(D)** Fasting blood glucose. **(E)** Urinary NAG. **(F)** Serum creatinine. **(G)** blood urea nitrogen. **(H)** Urinary albumin-to-creatinine. Results represent means ± SEM, *n* = 4. *P < 0.05, **P < 0.01, ***P < 0.001. STZ, streptozotocin; i.p., intraperitoneal injection; DKD, diabetic kidney disease; Val, DKD + Valsartan treated; Sac/Val, DKD + Sacubitril/Valsartan treated; KW, kidney weight; BW, body weight; NAG, N-acetyl-β-d-glucosamine-dase; Scr, serum creatinine; BUN, blood urea nitrogen; ACR, albumin-to-creatinine

The experimental schedule was presented in Fig. 1A. After treatment of 12 weeks, the FBG, kidney weight-to-body weight ratio (KW/BW), serum BUN, and urinary NAG and ACR of DKD mice were significantly increased compared with the control group. The DKD mice treated with Val and Sac/Val showed statistically lower KW/BW compared with those treated with vehicle, and the BW loss induced by DM was alleviated in Val group and Sac/Val group (Fig. 1B, C). However, Sac/Val or Val did not change the FBG level of diabetic mice (Fig. 1D). Interestingly, Sac/Val-treated DKD mice had lower Scr, serum BUN and urinary NAG compared with DKD group, while these parameters were not significantly lower than DKD group in Val group (Fig. 1E-G). Further, we also found that both Sac/Val and Val treatment significantly lowered 24-hour urinary ACR of DKD mice, but there were no statistical differences between Val and Sac/Val group (Fig. 1H). These data suggest that Sac/Val has markedly renoprotective effects.

### Sacubitril/valsartan attenuates tubulointerstitial fibrosis in DKD mice

**Fig. 2:**
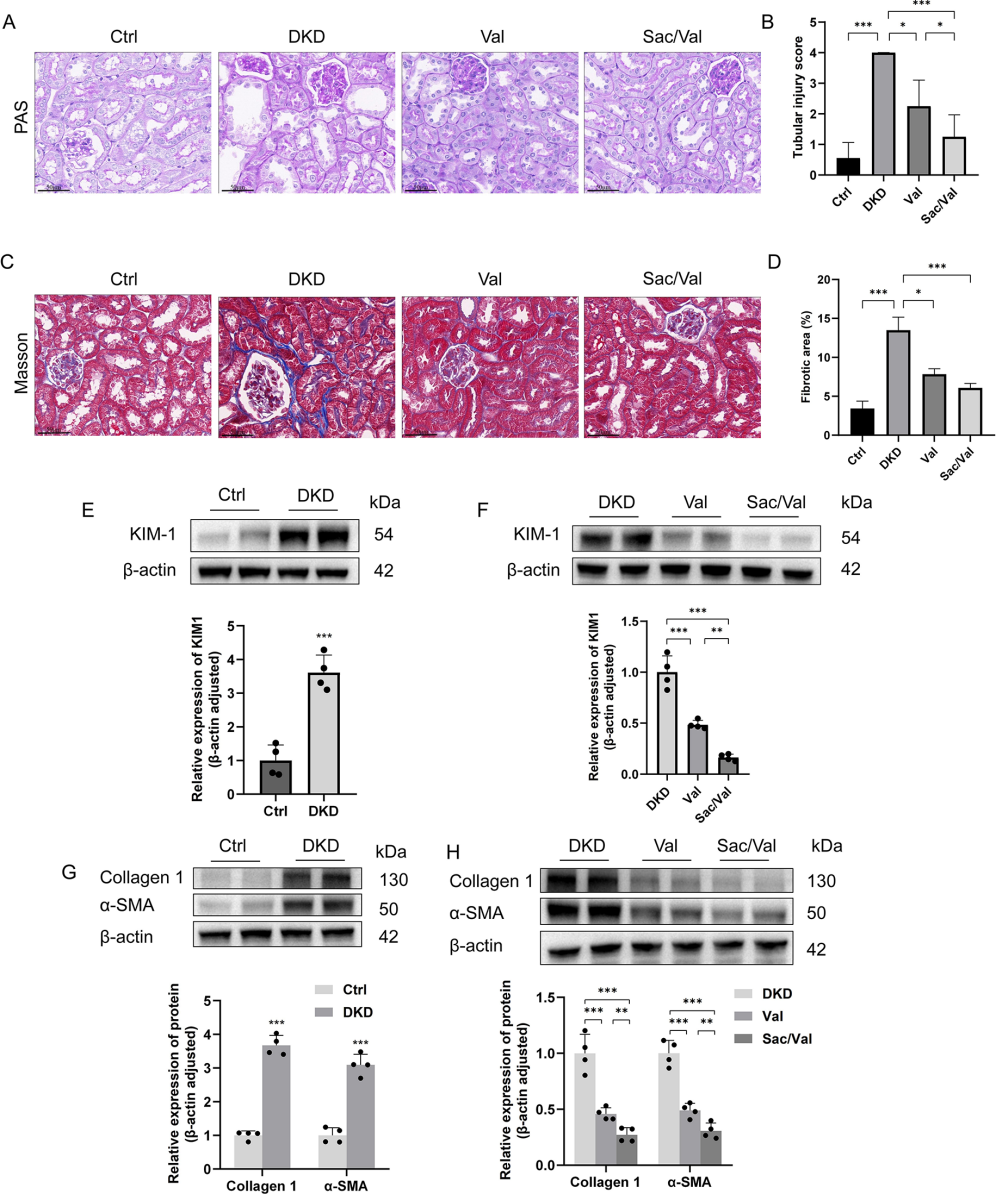
Sacubitril/valsartan attenuated tubulointerstitial fibrosis in diabetic mice. **(A)** PAS staining, representative micrographs were shown. **(B)** Tubular injury score in PAS-stained sections. **(C)** Masson staining, representative micrographs were shown. **(D)** The percentage of tubulointerstitial fibrotic area in Masson trichrome-stained sections. **(E, F)** Representative western blotting images and densitometric analysis of KIM-1. **(E, G)** Representative western blotting images and densitometric analysis of Collagen 1 and α-SMA. Scale bar = 50 μm. Results represent means ± SEM, *n* = 4. *P < 0.05, **P < 0.01, ***P < 0.001. DKD, diabetic kidney disease; Val, DKD + Valsartan treated; Sac/Val, DKD + Sacubitril/Valsartan treated; PAS, periodic acid-Schiff base

Histologically, loss of the brush border, tubular dilation, tubular atrophy, cast formation, sloughing of tubular epithelial cells and thickening of the tubular basement membrane were significantly attenuated in DKD mice with Sac/Val treatment (Fig. 2A). Meanwhile, we observed that tubulointerstitial fibrosis in DKD mice was significantly increased. While it was markedly attenuated with Sac/Val treatment, evidenced by the results of Masson staining (Fig. 2C). Concomitantly, according to the results of quantitative assessment, the effects of Sac/Val on reducing tubular injury score and tubulointerstitial fibrotic area were superior to that of Val (Fig. 2B, D). Further, the protein expression of KIM-1 (a specific and sensitive biomarker of tubule injury) was significant attenuated by Sac/Val (Fig. 2E-F). The protein expression of collagen 1 and α-SMA in the kidney followed a similar trajectory (Fig. 2G-H). Collectively, these data indicated that Sac/Val attenuates renal tubular injury and tubulointerstitial fibrosis.

### Sacubitril/valsartan restores mitochondrial homeostasis

**Fig. 3:**
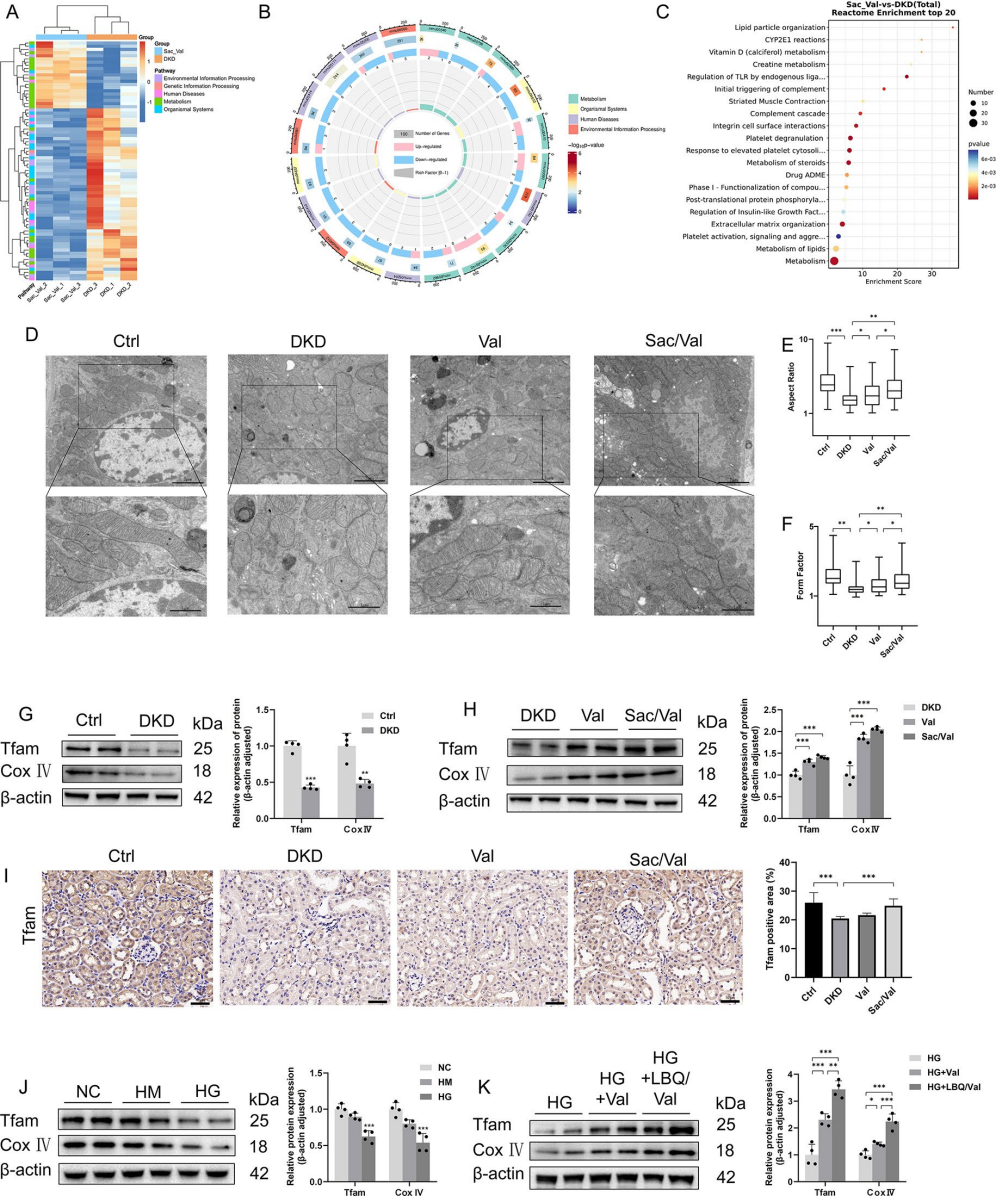
Sacubitril/valsartan improved mitochondrial morphology and mitochondrial biogenesis. **(A-C)** Functions of differential expressed genes between DKD group and Sac/Val group **(D)** Representative transmission electron microscopy of mitochondria in tubular cells of mice. **(E)** Aspect ratio of mitochondria in tubular epithelial cells. **(F)** Form factor of mitochondria in tubular epithelial cells. **(G, H)** Representative western blotting images and densitometric analysis of Tfam and Cox IV; in kidney from mice in different groups. **(I)** Representative images and statistical graphs of immunohistochemical staining of Tfam in kidney sections. **(J, K)** Representative western blotting images and densitometric analysis of Tfam and Cox IV; in HK-2 cells. Scale bar = 50 μm. Results represent means ± SEM, *n* = 4. *P < 0.05; **P < 0.01; ***P < 0.001 DKD, diabetic kidney disease; Val, DKD + Valsartan treated; Sac/Val, DKD + Sacubitril/Valsartan treated; NC, normal control; HM, high mannitol; HG, high glucose; HG + Val, high glucose + Valsartan; HG + LBQ/Val, high glucose + LBQ657 + valsartan

To explore the potential mechanisms of Sac/Val on the amelioration of tubulointerstitial fibrosis, a genome-wide gene expression analysis of kidney tissues was performed. The up-regulated and down-regulated genes displayed by Heatmap (Fig. 3A). To identify the inherent transcriptome features and predict the function of differentially expressed genes, we applied KEGG (Fig. 3B) and Reactome analyses (Fig. 3C) for feature selection. Considering the function of Sac/Val on the amelioration of tubulointerstitial fibrosis, we focused on the differentially expressed genes that were specifically downregulated. Interestingly, the downregulated expressed genes were found to be associated with organismal systems, metabolism, and environmental information processing.

Next, tubular mitochondrial homeostasis was examined. Interestingly, TEM revealed more fragmented mitochondria with decreased AR and FF in DKD mice, which was markedly alleviated by Val or Sac/Val treatment (Fig. 3D). Meanwhile, we found that, compared to the mice with Val treatment, the improvement of AR and FF was more significant in mice with Sac/Val administration (Fig. 3E, F). Further, we analyzed mitochondria function by detecting the Cox IV; (a subunit of electron transport chain proteins regulating cellular energy metabolism) and Tfam (a mitochondrial protective protein that promotes replication and transcription of mitochondrial DNA). Compared to the control group, diabetic mice showed significantly lower levels of Cox IV; and Tfam protein expression, which was largely restored by Sac/Val (Fig. 3G-H). As shown in Fig. 3I, the immunohistochemical staining analysis also revealed increased Tfam expression in the kidney tissues of DKD mice treated with Sac/Val.

**Fig. 4:**
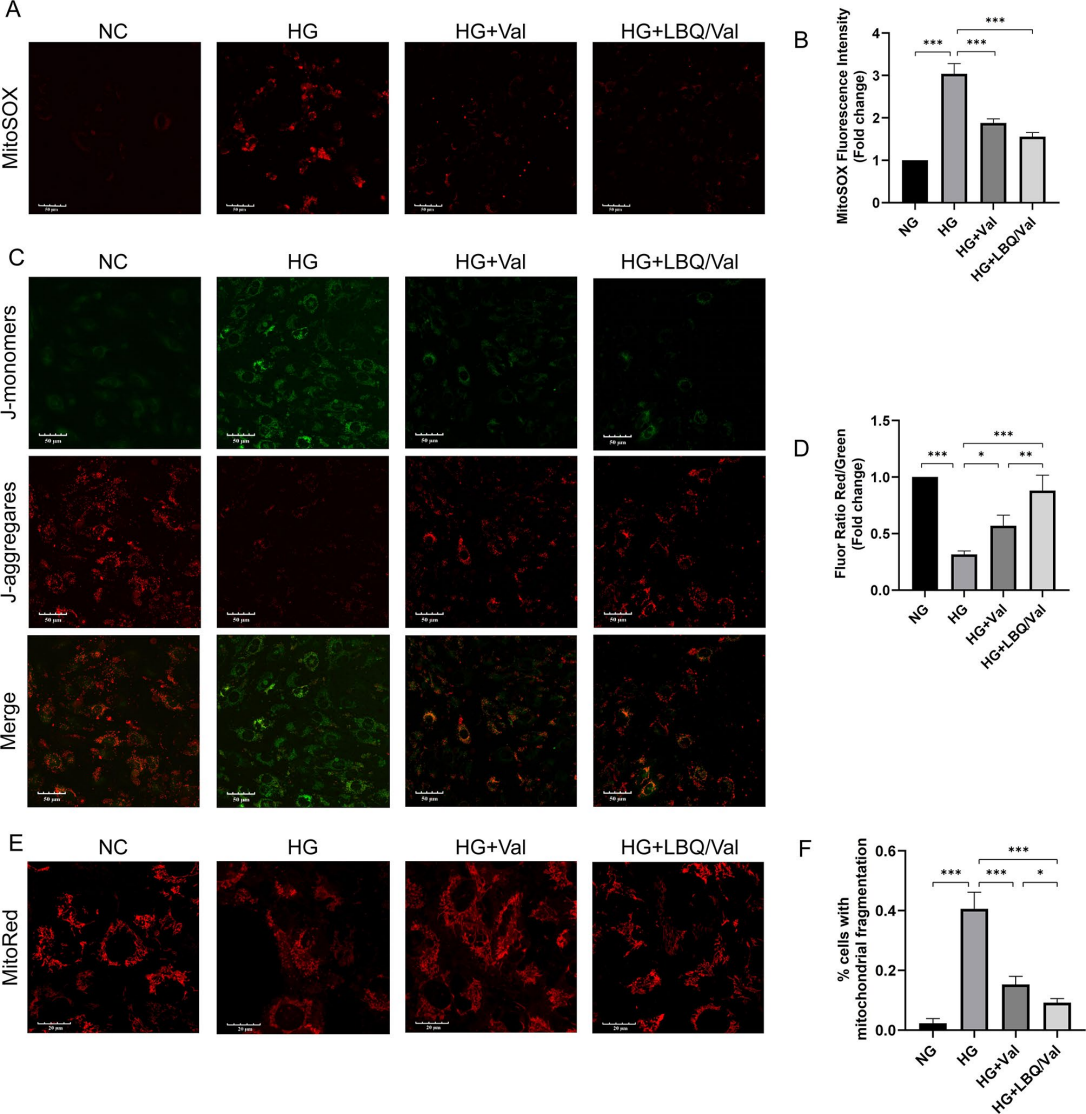
In vitro sacubitril/ valsartan improved mitochondrial function and morphology in HG-treated HK-2 cells. **(A**, **B)** Typical fluorescence photomicrograph and quantitative analysis of mitochondrial superoxide. **(C**, **D)** Mitochondrial membrane potential assessed by JC-1 assay. **(E)** Representative images of mitochondria morphology with MitoTracker staining. **(F)** The percentage of cells with mitochondrial fragmentation in MitoTracker staining. Results represent means ± SEM, *n* = 4. *P < 0.05, **P < 0.01, ***P < 0.001. NC, normal control; HM, high mannitol; HG, high glucose; HG + Val, high glucose + Valsartan; HG + LBQ/Val, high glucose + LBQ657 + valsartan

Thereafter, the potential function of Sac/Val in HG HK-2 cell was determined. Interestingly, we found that LBQ/Val, the active form of Sac/Val, increased the expression of Tfam and Cox IV, which was decreased by HG administration in HK-2 cells (Fig. 3J-K). Of note, although both Val and LBQ/Val improved expressions of these proteins, LBQ/Val had relatively stronger effects than Val. Then, mitochondrial ROS, MMP and mitochondrial morphology in HK-2 cells were assessed. Hyperglycemia increased MitoSOX fluorescence intensity of HK-2 cells, indicating the increased in mitochondrial ROS production, while LBQ/Val markedly attenuated MitoSOX expression in HG-treated HK-2 cells (Fig. 4A-B). Compared to the NC group, JC-1 staining showed increased green fluorescence and weaker red fluorescence in the cells with HG administration, revealing the reduction of MMP under HG conditions. Administration of LBQ/Val resulted in amelioration of aberrant MMP (Fig. 4C-D). Finally, the fragmented mitochondria assessed by MitoTracker in the HK-2 cells followed a similar pattern (Fig. 4E-F). Thus, these data indicate that Sac/Val improved mitochondrial dysfunction in DKD.

### Sacubitril/valsartan activated Sirt1/PGC1α pathway

**Fig. 5:**
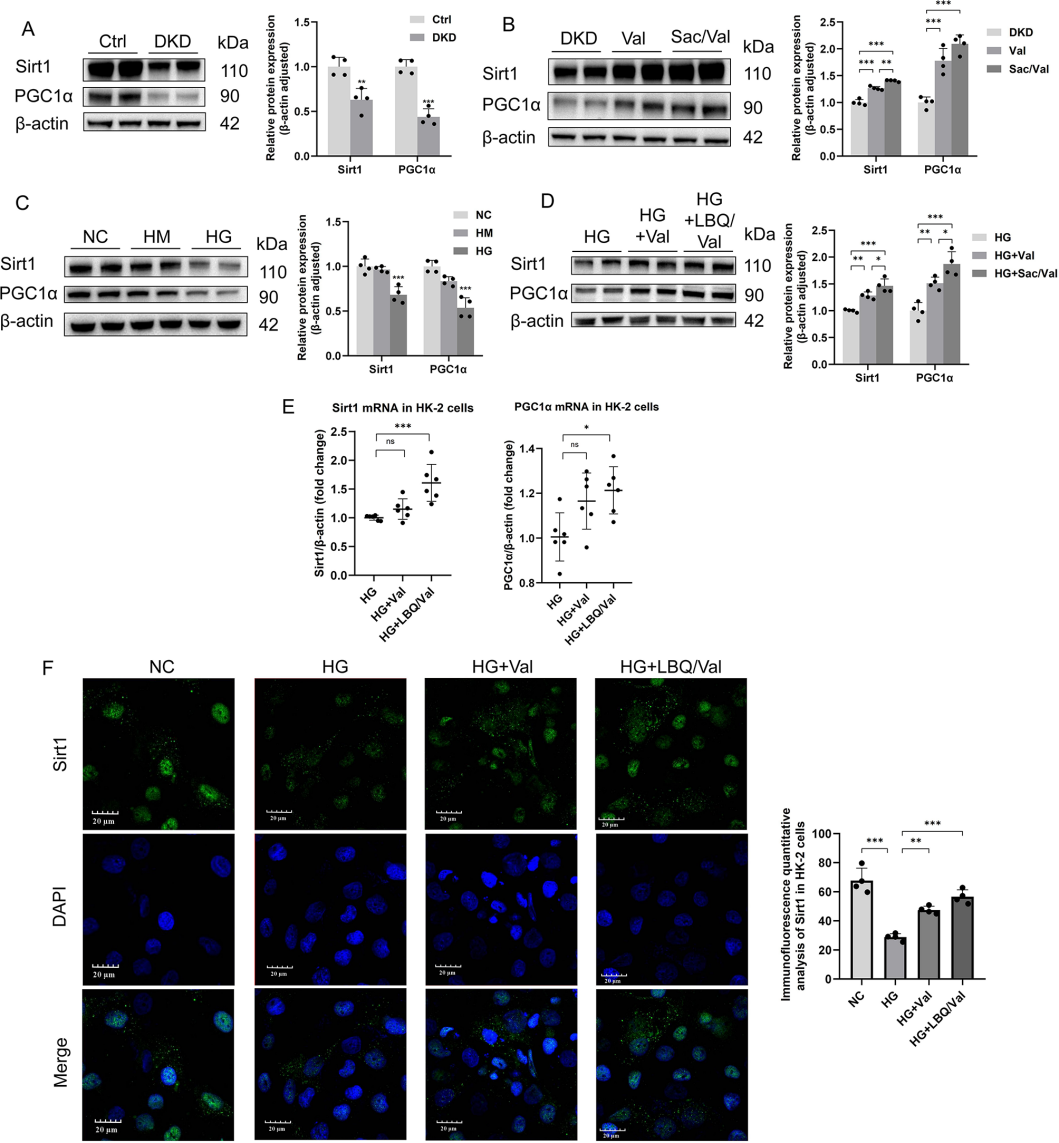
Sacubitril/valsartan improved expression of Sirt1/PGC-1α in DKD mice and HG HK-2 cells. **(A**, **B)** Representative western blotting images and densitometric analysis of Sirt1 and PGC1α in kidney from mice. **(C**, **D)** Representative western blotting images and densitometric analysis of Sirt1 and PGC1α in HK-2 cells. **(E)** PCR analysis of Sirt1 and PGC1α mRNA expression in HK-2 cells treated with HG. **(F)** Representative images and statistical graphs of immunofluorescent staining for Sirt1 in HK-2 cells from different groups. Results represent means ± SEM, *n* = 4. *P < 0.05, **P < 0.01, ***P < 0.001. DKD, Diabetic kidney disease; Val, DKD + Valsartan treated; Sac/Val, DKD + Sacubitril/Valsartan treated; NC, normal control; HM, high mannitol; HG, high glucose; HG + Val, high glucose + Valsartan; HG + LBQ/Val, high glucose + LBQ657 + valsartan

Previous studies indicated that PPAR-γ coactivator-1α (PGC1α), which can be activated via the deacetylation by Sirtuin1 (Sirt1), plays a vital role in mitochondrial homeostasis [18, 19]. Compelling evidence showed that activation of Sirt1 and PGC1α restores mitochondrial function and biogenesis and played an essential role in compensatory response of mitochondrial homeostasis [20]. Considering the key role of Sirt1/PGC1α axis in the regulation of mitochondrial homeostasis, we hypothesized that mitochondrial homeostasis-mediated by Sirt1/PGC1α axis is the exact molecular mechanism for the renoprotective effects of Sac/Val. Interestingly, the decreased expressions of Sirt1 and PGC1α in DKD mice were improved by Sac/Val (Fig. 5A, B). As in DKD mice, the expressions of Sirt1 and PGC1α were markedly decreased in HK-2 cells of HG group (Fig. 5C). The results of Western blot and real-time PCR revealed that LBQ/Val improved protein and mRNA expression of Sirt1 and PGC1α, suggesting that the Sirt1/PGC1α pathway was activated by LBQ/Val (Fig. 5D, E). Further, the immunofluorescence staining for Sirt1 in the kidney followed a similar pattern (Fig. 5F). The results all suggested that there is a potential link between the renoprotective effect of Sac/Val and Sirt1/PGC1α pathway in DKD.

### Sacubitril/valsartan restored mitochondrial homeostasis via Sirt1/PGC1α pathway

**Fig. 6:**
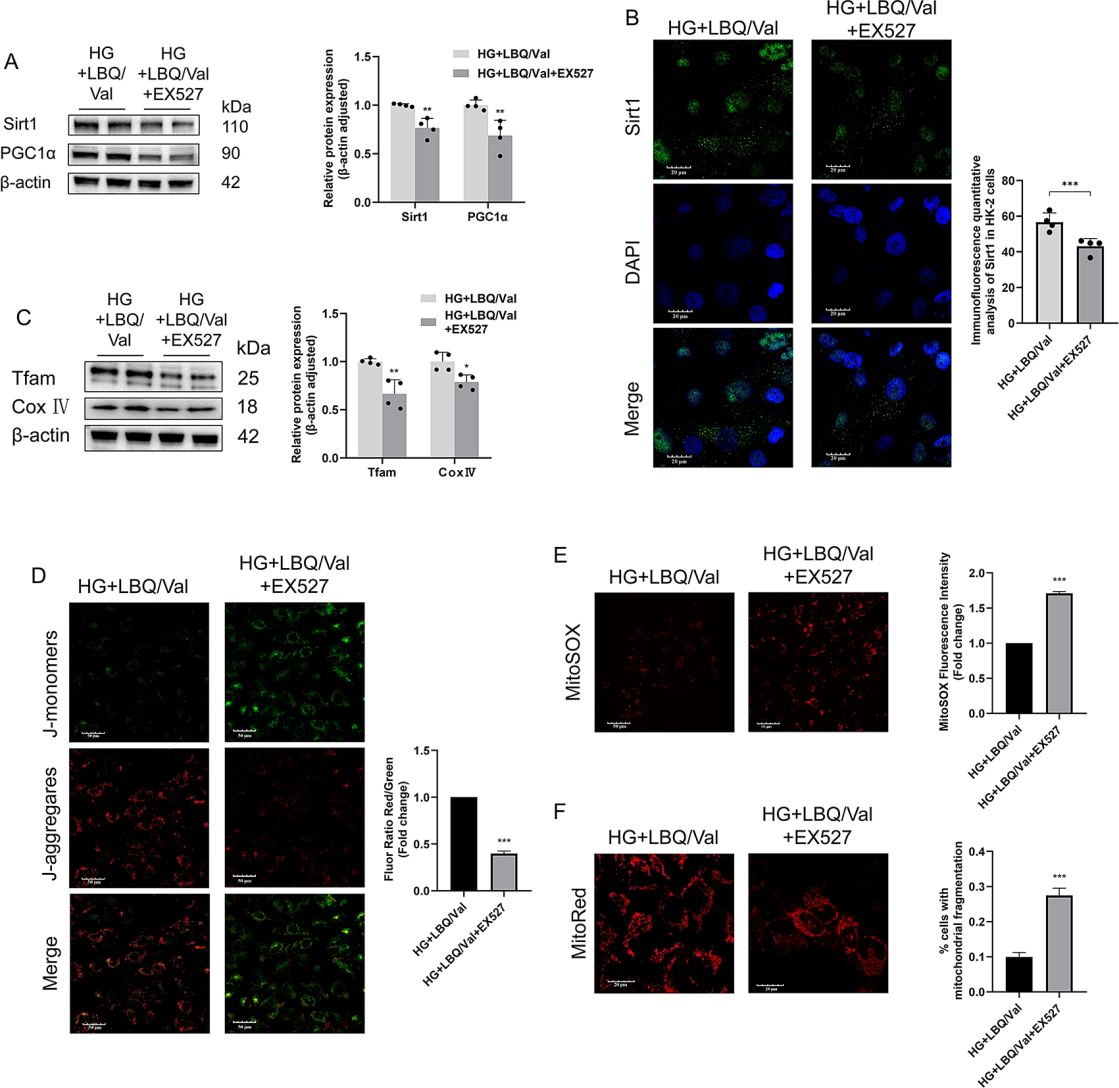
The improvement of mitochondrial function by in Vitro sacubitril (LBQ657) /valsartan was attenuated by Sirt1 inhibition in the HG-treated HK-2 cells. **(A)** Representative western blotting images and densitometric analysis of Sirt1 and PGC1α of HK-2 cells from HG + LBQ/Val and HG + LBQ/Val + EX527 group. **(B)** Representative images and statistical graphs of immunofluorescent staining for Sirt1. **(C)** Representative Western blotting images and densitometric analysis of Tfam and Cox IV;. **(D)** Mitochondrial membrane potential assessed by JC-1 assay. **(E)** Typical fluorescence photomicrograph and quantitative analysis of mitochondrial superoxide. **(F)** Representative images of mitochondria morphology with MitoTracker staining and quantitative analysis of the percentage of cells with mitochondrial fragmentation. Results represent means ± SEM, *n* = 4. *P < 0.05, **P < 0.01, ***P < 0.001. HG, high glucose; Val, valsartan; LBQ/Val, LBQ657 + valsartan

The Sirt1 specific inhibitor, EX527, markedly reduced the expressions levels of Sirt1 and PGC1α in the HK-2 cells with LBQ/Val treatment under HG conditions (Fig. 6A, B). The effects of LBQ/Val on mitochondrial function were also weaken after Sirt1 was inhibited. Protein expression levels of Tfam and Cox IV; were lower in HG + LBQ/Val + EX527 group than LBQ/Val group (Fig. 6C). EX527 partially reversed the improvement of MMP and exacerbated the production of mitochondrial ROS in the cells treated by LBQ/Val (Fig. 6D, E). Meanwhile, the HK-2 cells of HG + LBQ/Val + EX527 group showed inferior improvement in mitochondrial morphology than HG + LBQ/Val group (Fig. 6F). Taken together, it can be speculated that the beneficial effects of Sac/Val are partially attributed to the activation of Sirt1/PGC1α pathway.

### Sacubitril/valsartan attenuated tubulointerstitial fibrosis through Sirt1/PGC1α pathway-mediated mitochondrial function

**Fig. 7:**
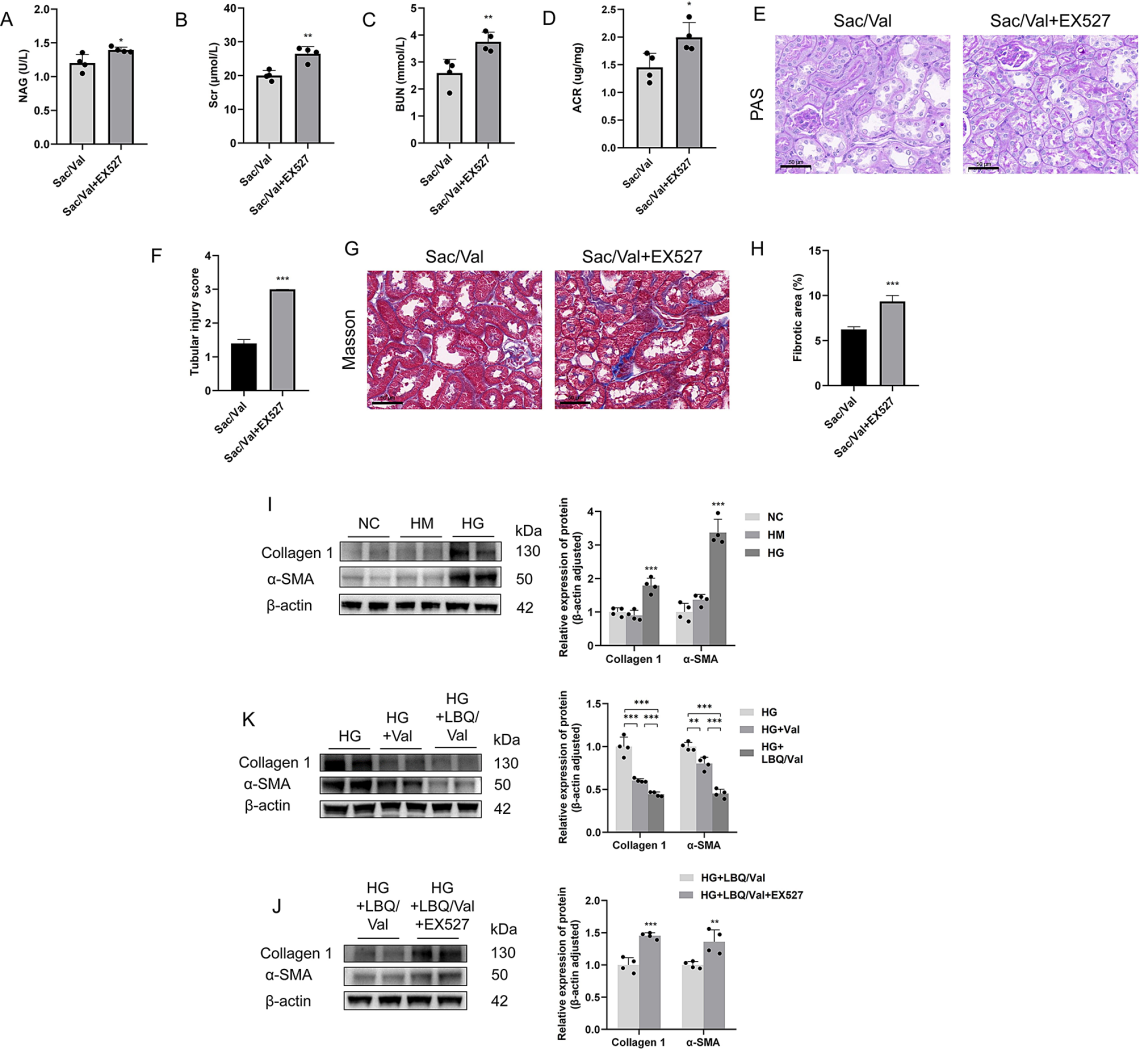
Sacubitril/valsartan attenuated tubulointerstitial fibrosis through Sirt1-mediated mitochondrial dysfunction. **(A)** Urinary NAG. **(B)** Serum creatinine. **(C)** blood urea nitrogen. **(D)** Urinary albumin-to-creatinine. **(E)** PAS staining, representative micrographs were shown. **(F)** Tubular injury score in PAS-stained sections. **(G)** Masson staining, representative micrographs were shown. **(H)** The percentage of tubulointerstitial fibrotic area in Masson trichrome-stained sections. **(I-K)** Representative Western blotting images and densitometric analysis of collagen 1 and α-SMA in HK-2 cells. Scale bar = 50 μm. Results represent means ± SEM, *n* = 4. *P < 0.05, **P < 0.01, ***P < 0.001. Sac/Val, DKD + Sacubitril/Valsartan treated; Sac/Val + EX527, DKD + Sacubitril/Valsartan and EX527 treated; NC, normal control; HM, high mannitol; HG, high glucose; HG + Val, high glucose + Valsartan; HG + LBQ/Val, high glucose + LBQ657 + valsartan

Our results of biochemistry and histopathological tests of DKD mice revealed that the renal function and tubulointerstitial fibrosis improved by Sac/Val was partially attenuated when Sirt1 was inhibited. Urinary NAG, Scr, serum BUN and urinary ACR was augmented in Sac/Val + EX527 group compared to those in Sac/Val group, indicating the relatively severe renal tubular injury and renal dysfunction (Fig. 7A-D). After treatment with EX527, the tubulointerstitial fibrosis that alleviated by Sac/Val was aggravated (Fig. 7E-H). When Sirt1 was inhibited, the effect of LBQ/Val on the alleviation of tubulointerstitial fibrosis was attenuated, indicated by the relatively high levels of collagen 1 and α-SMA (Fig. 7I-K). Therefore, it can be inferred that mitochondrial function regulated by Sirt1/PGC1α is involved in the mechanism by which Sac/Val ameliorates tubulointerstitial fibrosis in DKD mice.

## Discussion

Recently, Sac/Val is found to exert a robust beneficial effect in DKD. However, the potential functional effect of Sac/Val on tubulointerstitial fibrosis is still largely unclear. Accordingly, in this study, we found that Sac/Val improved renal function and attenuated tubulointerstitial fibrosis through restoring the disturbed mitochondrial homeostasis. Further, activation of Sirt1/PGC1α pathway, which is mediated by Sac/Val, has been proven to be the crucial mechanism. Therefore, our finding provides a theoretical basis for delaying the progression of DKD in clinical practice.

Previously, growing evidence indicated that Sac/Val could prevent worsening of renal function and progression of chronic kidney disease (CKD). For instance, Spannella et al. reported that Sac/Val resulted in a lower risk of renal dysfunction as compared with RAS inhibitors alone, evidenced by a systematic review and meta-analysis of 10 randomized controlled trials [21]. Meanwhile, preclinical study also revealed that Sac/Val improved the decline of renal function in DKD mice [14]. However, the underlying mechanism is still unclear.

Therefore, the exact effects and molecular mechanism of ARNI on renal function in DKD was explored. Interestingly, here, we found that Sac/Val effectively improved decline of renal function of diabetic mice, demonstrated by the reduced Scr and BUN in Sac/Val group. This is in line with the studies focusing on the renoprotective effects of Sac/Val in diabetic animal models [22, 23] and other kidney disease models [24–26]. Meanwhile, urinary NAG was also lower in Sac/Val group compared with that in DKD group and Val group, indicating that Sac/Val was superior to Val on alleviating tubular injury in DKD mice. Concomitantly, we found that Sac/Val significantly alleviated tubulointerstitial fibrosis, which is one of the most important findings of this study. In addition, the reduction in urinary ACR suggested that proteinuria, which may aggravate the production of proinflammatory and profibrotic factors [27], was attenuated by Sac/Val. Of note, there was no significant change in FBG after treatment with Val or Sac/Val, thus, the renoprotection effects of Sac/Val may contribute to the attenuated tubule injury.

What is the exact mechanism of renoprotection effects of Sac/Val? Emerging evidence indicated that tubular injury and tubular dysfunction occur in the early stage of DKD and plays a critical role in the progression of DKD. Recent evidence also suggested that tubulointerstitial damage may start from a primary tubular injury initiated by metabolic disorder [28]. Packed with mitochondria and dependent on oxidative phosphorylation, the proximal tubule is particularly vulnerable to injury (hypoxic, oxidative, metabolic), resulting in mitochondrial disorders [29, 30]. When exposed to HG environments, RTECs need a large amount of ATP to reabsorb excess glucose, resulting in superoxide production concurrently [30]. Excessive superoxide is converted into ROS, which contributes to mitochondrial damage, thereby leading to atrophy or programmed cell death of RTECs. Therefore, increasing evidence suggests that mitochondrial dysfunction is an important pathological factor promoting tubulointerstitial fibrosis in kidney diseases, including DKD [8, 31].

In our study, both in vivo and in vitro experiment showed that the decreased expressions of Tfam and Cox IV; in DKD were effectively attenuated by Sac/Val, indicating the improvement of mitochondrial biogenesis. The results of JC-1, MitoSOX and MitoTracker assessment also revealed the beneficial effects of Sac/Val on mitochondrial function. The improved mitochondrial function may contribute to Sac/Val’s protection effects on tubular injury. Furthermore, it has been extensively shown that high glucose levels in diabetes increases Ang II expression, which induces cellular hypertrophy of tubular cells mediated by the activation of TGF-β [32, 33]. Therefore, inhibition of RAAS may be another mechanism by which Sac/Val exerts its protective effect on tubular cells of diabetic kidney.

Then, the molecular mechanism of Sac/Val on tubular mitochondrial homeostasis was explored. Compelling evidence has demonstrated that Sirt1 deacetylates multiple lysine sites of PGC1α to promote the reconstruction of cellular energy homeostasis [34]. This deacetylation of PGC1α results in a well-coordinated change of gene expression that related to transcriptional control of mitochondrial proteins, including PPARα, hepatocyte nuclear factor 4α, estrogen-related receptor α, Forkhead box-containing protein type O 1, nuclear respiratory factor [35, 36]. Moreover, targeting Sirt1/PGC1α has been considered to be a promising strategy to prevent the progression of DKD. Here, we observed that the decreased Sirt1 and PGC1α expression, as well as the aberrant mitochondrial morphology and function in DKD mice and HG-treated HK-2 cells were markedly ameliorated by Sac/Val. More interestingly, the functional effects of Sac/Val on mitochondria were dependent on the Sirt1/PGC1α signaling. Because after treatment with Sirt1 specific inhibitor EX527, the alleviated mitochondrial abnormalities and tubulointerstitial fibrosis by Sac/Val was markedly reversed.

In fact, the functional roles of Sac/Val in mitochondria and metabolism in patients has been observed. For instance, Selvaraj et al. reported that compared with valsartan, Sac/Val reduced triglycerides and increased high-density lipoprotein cholesterol [37]. Meanwhile, in the diabetic sub-study of the PARADIGM-HF trial, sacubitril/valsartan administration was also found to increase high-density lipoprotein cholesterol level [38]. These data suggested that Sac/Val plays a vital role in regulating of metabolism, which is a key factor in regulating renal tubule injury and renal fibrosis [39]. Furthermore, in the PARAGON-HF trial, Sac/Val showed superior outcome of renal function in patients compared to Val [40], which is consistent with our findings. To the best of our knowledge, our study is the first to explore the role of Sac/Val in mitochondria and metabolism under the condition of DKD.

Finally, the exact mechanisms of Sac/Val on Sirt1/PGC1α pathway activation remains unclear. Nevertheless, we speculate that Sac/Val may potentiate Sirt1/PGC1α pathway by enhancing the natriuretic peptides (NPs)-cGMP-dependent pathway. Because cGMP-dependent pathway was found to increase the level and activity of Sirt1, which is associated with a decrease in the activity of NADPH oxidase and the levels of ROS [41]. Moreover, it has been widely accepted that cGMP level was decreased in diabetic kidney [42]. Whereas, Sac/Val contributed to an increase in the level of NPs by inhibiting NPs degradation, which activates its guanylyl cyclase (GC)-A through binding to NPR-A and subsequently elevates the intracellular level of cGMP [43]. Of note, Jani´c et al. reported that the administration of Val substantially increased the expression of Sirt1 in healthy middle-aged males [44]. Therefore, we hypothesize that the effects of Sac/Val on Sirt1/PGC1α pathway may be cGMP-dependent.

## Conclusions

Taken together, we demonstrated that Sac/Val ameliorates tubulointerstitial fibrosis in DKD. Mechanistically, Sac/Val could restore disturbed mitochondrial homeostasis in tubules through activating Sirt1/PGC1α pathway. Therefore, our findings provide a theoretical basis for delaying the progression of DKD.

## Data Availability

The data used and analyzed during the current study are available from the corresponding author on reasonable request.

## Abbreviations

DKD: Diabetic kidney disease
Sirt1: Sirtuin1
PGC1α: Peroxisome proliferator-activated receptor-γ coactlvator-1α
HG: High glucose
DM: Diabetes mellitus
RTECs: Renal tubular epithelial cells
ATP: Adenosine triphosphate
CKD: Chronic kidney disease
RAAS: Renin-angiotensin aldosterone system
Sac/Val: Sacubitril/Valsartan
Val: Valsartan
ARNI: Angiotensin receptor-neprilysin inhibitor
ARB: Angiotensin II receptor blocker
cGMP: Cyclic guanosine monophosphate
STZ: Streptozotocin
FBG: Fasting blood glucose
BW: Body weight
KW: Kidney weight
HG: High glucose
HM: High mannitol
NC: Normal control
Scr: Serum creatinine
BUN: Blood urea nitrogen
NAG: N-acetyl-β-d-glucosamine-dase
ACR: Albumin-to-creatinine ratio
PAS: Periodic acid-Schiff base
TEM: Transmission electron microscopy
AR: Aspect ratio
FF: Form facto
Tfam: Mitochondrial transcription factor A
KIM-1: Kidney injury molecule-1
α-SMA: α-Smooth muscle actin
MMP: Mitochondrial membrane potential
ROS: Reactive oxygen species
SEM: Standard error
TGF-β: Transforming growth factor β
NPs: Natriuretic peptides
NPR-A: Natriuretic peptide receptor-A
GC: Guanylyl cyclase
